# Surgical outcome of jejunum-jejunum intussusception secondary to Rapunzel syndrome: a case report

**DOI:** 10.1186/s13256-018-1883-9

**Published:** 2018-12-07

**Authors:** Martín Adrián Bolívar-Rodríguez, Rodolfo Fierro-López, Adrián Pamanes-Lozano, Marcel Antonio Cazarez-Aguilar, Benny Alonso Osuna-Wong, José Cándido Ortiz-Bojórquez

**Affiliations:** Departament of General Surgery, Centro de Investigación y Docencia en Ciencias de la Salud de la Universidad Autónoma de Sinaloa en el Hospital Civil de Culiacán, Culiacán, Mexico

**Keywords:** Intestinal intussusception, Rapunzel syndrome, Trichobezoar, Gastrotomy

## Abstract

**Background:**

Adult intestinal intussusception is a rare condition caused by the mechanical disruption of bowel motility. A bezoar is defined as indigestible material inside the gastrointestinal tract that develops into a trapped mass; the most frequent bezoar is a trichobezoar. When a trichobezoar extends into the small intestine it is defined as Rapunzel’s syndrome. Literature describing complications related to this pathology remains scarce.

**Case presentation:**

A 16-year-old Mexican girl presented to our emergency room with acute abdomen and a presumptive diagnosis of intestinal obstruction. Computed tomography was suggestive of intussusception. Surgery confirmed a jejunal-jejunal intussusception with a mass within the gastric cavity extending into her small intestine, corresponding to a trichobezoar. A manual intussusception reduction and a gastrotomy with extraction of the trichobezoar were performed.

**Conclusions:**

We present a case of a jejunum intussusception as a complication of Rapunzel syndrome. Our patient had a favorable outcome after surgical intervention with a manual intussusception reduction, with retrograde displacement of the trichobezoar into the gastric lumen, and a complete extraction through a gastrostomy. Follow-up included psychiatric evaluation.

## Background

Adult intestinal intussusception is a rare condition caused by a mechanical disruption of motility due to an infection, tumor, Meckel’s diverticulum, and intestinal duplication among others [1]. A bezoar is defined as undigested material inside the gastrointestinal tract that eventually becomes a trapped mass. Trichobezoars are made up of hair and are associated with psychiatric conditions such as trichophagia and trichotillomania [2]. Trichobezoars are usually progressive in nature and begin in the stomach [3]. Trichobezoars are the most frequent type of bezoar in patients under 30 years of age [4]. Baudamant was the first to report a trichobezoar in 1779 [5]. If the bezoar happens to extend into the small intestine, this is termed Rapunzel syndrome as described by Vaughan *et al.* in 1968 [6]. Rapunzel syndrome presents with symptoms of intestinal obstruction in which there is a gastric trichobezoar with a “tail-like” post-pyloric extension into the small intestine. The diagnosis is confirmed by surgical findings [7].

Most published case reports focus on intestinal obstruction or perforation [2]. However, a few discuss the relationship between intussusception and Rapunzel syndrome [1], as we report in the following case.

## Case presentation

A 16-year-old Mexican girl presented with a 4-day history of epigastric abdominal pain that radiated to the left hypochondrium and was accompanied by abdominal bloating. She reported vomiting approximately 30 times 24 hours after symptom onset. Oral intake of fluids and solid food was impaired, and both flatus and bowel movements were absent. She had no history of prior surgical interventions and did not have a history of fever, hematemesis, jaundice, chyluria, or acholic stools.

She was hemodynamically stable on room air with a mild tachycardia of up to 140 beats per minute (bpm). On physical examination she presented no neurological alterations or alopecia. An abdominal examination revealed distention, borborygmi, painful palpation, and involuntary resistance in upper quadrants with rebound tenderness.

**Fig. 1:**
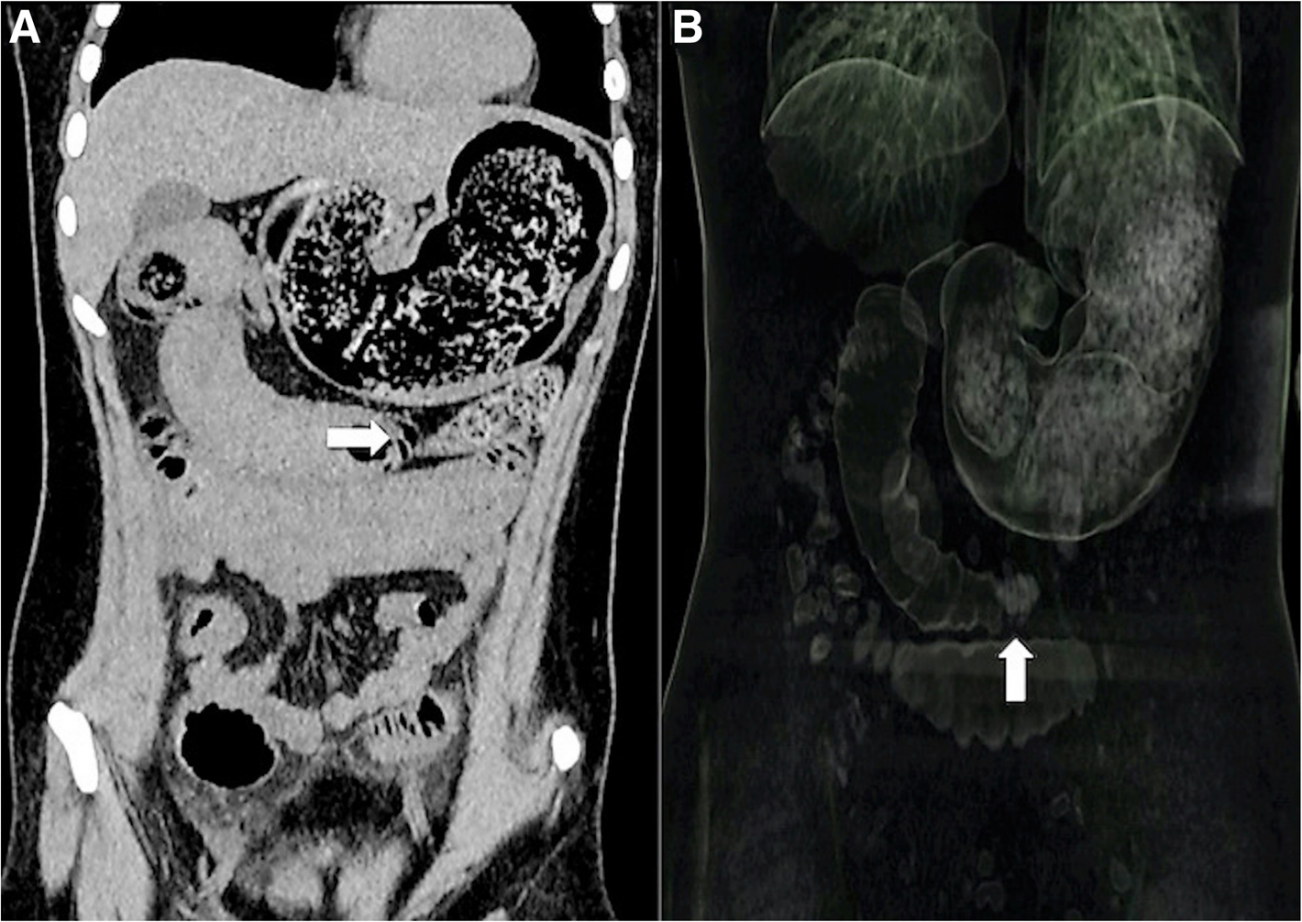
**a** Computed tomography with coronal reconstruction, we observe a beehive pattern within the gastric chamber that extends into duodenum, observing a transition zone (*arrow*) in the proximal jejunum. **b** Volumetric reconstruction, observing an image suggesting intussusception (*arrow*)

She was hemoconcentrated with a hematocrit of 40 and had leukocytosis of 17,560/mm^3^. A computed tomography of her abdomen and pelvis with intravenously and orally administered contrast showed dilatation of the gastric chamber with a hyperdense beehive pattern (Fig. 1a, b). Dilated small intestine loops with fluid levels and a target image in the jejunum were suggestive of intussusception. Furthermore, findings were compatible with a trichobezoar.

**Fig. 2:**
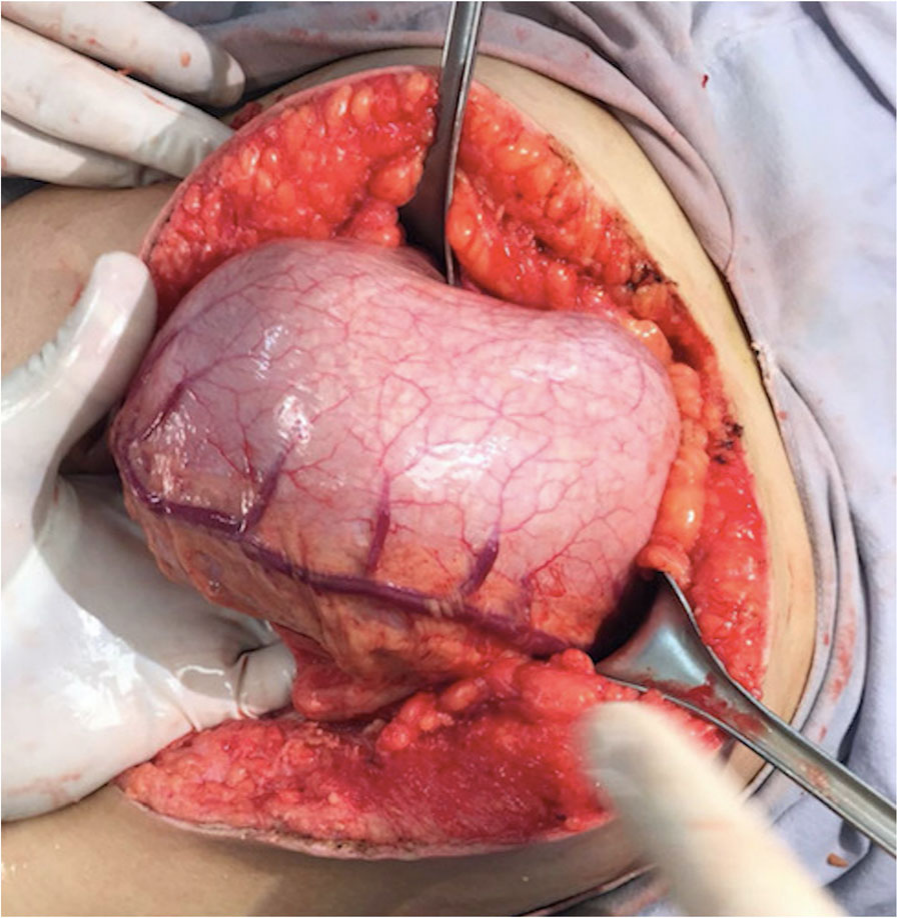
Anterior wall of the stomach observing dilated gastroepiploic arteries

**Fig. 3:**
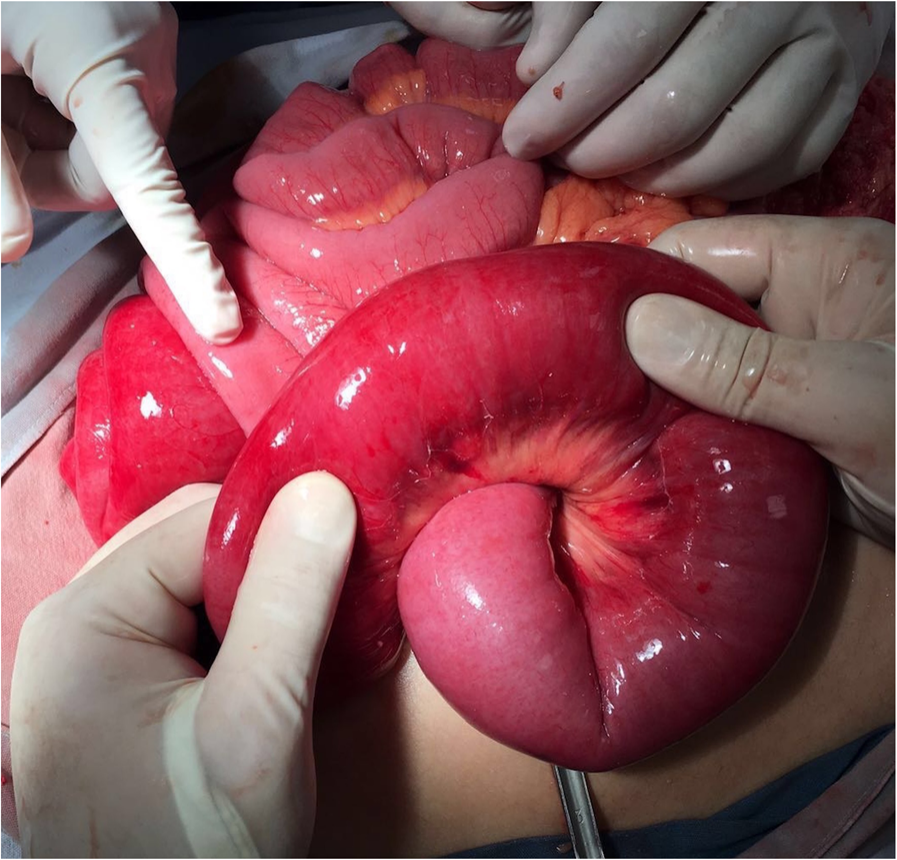
Jejunum loop with macroscopic changes (edema, swollen, hyperemia) due to intussusception

She underwent an exploratory laparotomy. Findings included gastric distention (Fig. 2), a palpable mass that extended from the gastric lumen to the first duodenum section, and a jejuno-jejunal intussusception (Fig. 3), which was liberated through manual revision. The jejunum showed macroscopic signs of inflammation 110 cm away from the ligament of Treitz. The mass was manually dragged into the duodenum.

**Fig. 4:**
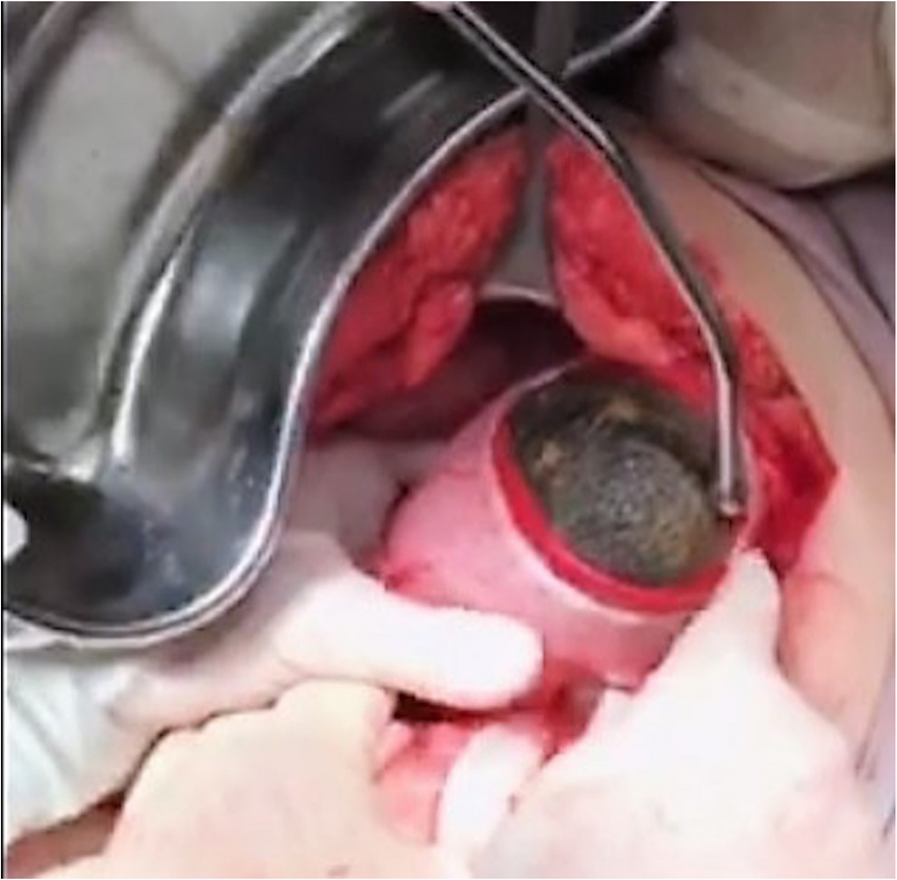
Trichobezoar extraction through gastrotomy from the anterior gastric wall with a longitudinal incision

**Fig. 5:**
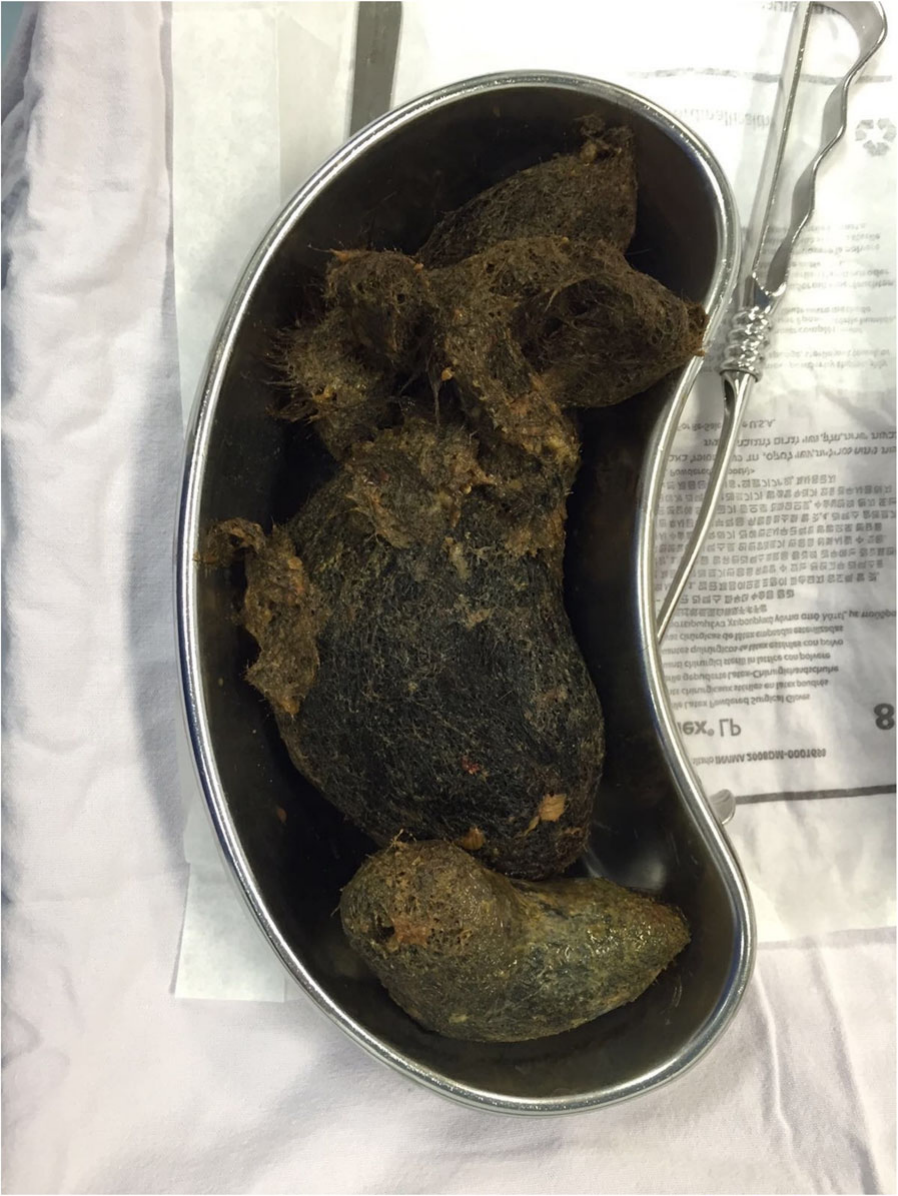
Extraction of trichobezoar segment

A 10 cm gastrotomy was performed on the anterior portion of the gastric body and a 20 cm-long continuous conglomerate of hair was extracted followed by four smaller fragments which extended to the site of the intussusception (Figs. 4 and 5). After gastric lavage with saline solution, a first intention closure was performed in two layers: first with polyglactin 00 using the Connell technique and then with gastric silk 00 using the Lembert technique. A 19 Fr closed system drainage was placed in her peritoneal cavity.

She had no immediate complications. Enteral nutrition was tolerated during the third postoperative day. Before hospital discharge she was evaluated by an in-house psychiatrist and was discharged during the fifth day of hospital admission.

## Discussion

Bezoars are classified into four groups: phytobezoar (indigestible fiber from fruits and vegetables), trichobezoar (hair), lactobezoar (milk protein/curd), and pharmacobezoar (medications) [8]. The term trichobezoar comes from the Greek word *trich*, which means hair, and *bāzahr*, which is Arabic for antidote [9]. Rapunzel’s syndrome is a rare form of trichobezoar, which receives its name due to its resemblance to a tail; it has a 1% incidence in patients with trichophagia [10]. In addition, 90% of cases involve women with underlying psychiatric disorders [11] and 10% involve young men [12].

Clinical presentation includes a palpable mass in the abdomen, abdominal pain, nausea, vomiting, weakness, weight loss, diarrhea, malnutrition, anorexia, and constipation depending on the degree of obstruction. The bezoar can cause a mass effect inside the gastrointestinal tract resulting in intestinal obstruction or gastric ulcer. The distal part of the trichobezoar (tail) may interfere with peristalsis and lead to an intussusception [13]. Patients are clinically diagnosed with intestinal obstruction. Medical evidence suggests a 6-month history of trichophagia is necessary in order to become symptomatic.

Image findings include an abdomen X-ray that shows a dilated gastric chamber with a complete filling image displacing intestinal loops. An abdominal ultrasound shows a highly reflective mass inside the stomach and duodenum with posterior acoustic shadowing. The most sensitive diagnostic study is computed tomography with double contrast; it shows gastric dilatation with a beehive pattern within that suggests trichobezoar, which extends to the duodenum.

A laparotomy is the surgical intervention of choice for elimination of the trichobezoar. Endoscopic removal remains controversial. Konuma *et al.* [14] describes a successful 15-minute endoscopic removal of a 34 cm, 100 g trichobezoar. However, a recent literature review by Gorter *et al.* [15] describes that only 5% of trichobezoars were susceptible for endoscopic extraction. Furthermore, conservative treatment with fizzy soft drinks has been described [16].

## Conclusions

Surgical management continues to be the treatment of choice for the resolution of a trichobezoar. Enterotomy is the preferred procedure for the extraction of a bezoar with intestinal extension. After extraction a systematic distal examination has to be performed in order to rule out a synchronic bezoar or a distal perforation.

In this case our patient presented Rapunzel syndrome with acute abdomen secondary to an occlusive jejunum-jejunum intussusception. The Rapunzel syndrome was surgically resolved with manual liberation, an anterograde retraction of the trichobezoar into the gastric lumen, and a complete extraction via gastrotomy. Follow-up treatment included psychiatric evaluation.
